# Address before the Pennsylvania State Medical Society

**Published:** 1857-11

**Authors:** 


ART. XI.—*An Address delivered before the Medical Society of the State of Pennsylvania, at its Annual Session, held in Westchester, in May,* 1857. By R. LA ROCHE, M.D., President of the Society; pp. 51.

AMONG the many annual addresses delivered at our medical colleges and before the numerous medical societies of the country, few, we venture to say, compare in point of genuine merit with the handsome production of Dr. La Roche. The name of the author is of itself a sufficient guarantee of the truth of this assertion, and while we would cheerfully give farther proof, by an extended analysis of the address, we are compelled, by want of room, to limit ourselves to the statement that it is a clear, manly exposition of the means by which alone medical science may be advanced, and the profession elevated to its proper rank. We advise our readers to procure the address, and to give it that careful and considerate perusal which it so justly claims.

